# Chemical Additives and Alternative Admixtures for Sustainable Construction Materials

**DOI:** 10.3390/ma19112232

**Published:** 2026-05-25

**Authors:** Zbyšek Pavlík, Milena Pavlíková

**Affiliations:** Department of Materials Engineering and Chemistry, Faculty of Civil Engineering, Czech Technical University in Prague, Thákurova 7, 166 29 Prague, Czech Republic; milena.pavlikova@fsv.cvut.cz

## Editorial Comment

The construction industry is under increasing pressure to reduce its environmental impact while simultaneously maintaining high levels of durability, safety, and performance in building materials. Cement and concrete production alone account for a significant proportion of global CO_2_ emissions, making the development of sustainable, low-carbon construction materials one of the most important research directions in contemporary materials science and civil engineering [1]. In this context, chemical additives and alternative admixtures represent highly promising tools for improving the functional properties, durability, and environmental efficiency of modern construction composites. Alternative low-carbon mixtures are designed through performance-based assessment combined with artificial intelligence techniques to optimise formulations alongside energy-efficient processes [2]. The machine learning-based approach incorporates multiple objective functions to optimise compressive strength, cost, and environmental impacts simultaneously, which enables the identification of various optimal mixture proportions that balance sustainability criteria and performance requirements effectively [3].

Chemical additives and mineral admixtures have long been essential for tailoring the properties of cementitious materials in both the fresh and hardened states, including workability, mechanical resistance, permeability, durability, and microstructural development [4]. The incorporation of supplementary cementitious materials, recycled resources, nanomaterials, and alternative binders, such as magnesia-based binders, also enables reductions in Portland clinker consumption and embodied carbon, while simultaneously enhancing the performance of construction materials [1,5]. Recently, there has been growing interest in graphene-based nanomodification, alkali-activated materials, low-carbon cement systems, and advanced multifunctional admixtures for sustainable construction applications [6,7,8,9]. These approaches support the principles of the circular economy and open up new possibilities for designing high-performance construction materials with lower environmental impacts. Magnesium-based cement, for example, is a viable alternative to traditional Portland cement and has the potential to reduce CO_2_ emissions [10]. Variants such as magnesium phosphate, magnesium sulphate, and magnesium oxychloride cement demonstrate excellent performance in certain applications. However, their air-hardening nature makes them unsuitable for water exposure, resulting in poor water resistance [11]. A more advanced, eco-friendlier alternative is the MgO–SiO_2_ (M–S) system, a hydraulic cement formed by the reaction of highly reactive magnesium oxide with silica sources in water. This reaction in the M–S system primarily results in the formation of magnesium silicate hydrate (M–S–H) gel, with magnesium hydroxide (Mg(OH)_2_) acting as an intermediate phase. The M–S system is characterised by low alkalinity, high strength, a large surface area, and strong corrosion resistance. These properties make it particularly suitable for applications in construction, nuclear waste treatment, heavy metal adsorption, refractory castables, and 3D printing, as well as in controlling C–S–H gel formation [12].

This Special Issue, entitled “Chemical Additives and Alternative Admixtures for Sustainable Construction Materials” was organised to present recent advances in the development, characterisation, and assessment of innovative construction materials modified with novel additives, admixtures, recycled resources, and advanced nanomaterials. The published papers cover a broad spectrum of sustainable material solutions, ranging from recycled polymer additives and alternative binders to graphene-modified composites and advanced insulation materials.

One important research direction represented in this Special Issue concerns the reutilisation of waste materials and recycled polymers in construction composites. Lee et al. [13] investigated the effect of waste PET fibres on the mechanical properties and chloride ion penetration resistance of emergency repair concrete for road pavements. Their results showed that recycled polymer fibres can significantly improve durability-related parameters while simultaneously contributing to waste reutilisation and sustainability in concrete technology. The use of recycled polymers and secondary raw materials in cementitious composites is increasingly recognised as an effective pathway toward low-carbon circular construction practices and a more sustainable built environment [14,15].

Similarly, Skowron et al. [16] studied the thermal and fire behaviour of rigid polyurethane (PUR) and polyisocyanurate (PIR) foams formulated with recycled PET polyols [17]. Their study contributes to the development of environmentally friendly insulation materials with improved thermal and fire resistance. Oumaima et al. [18] developed sustainable aerogel blankets from recycled plastics (PET fibres) and construction waste for high-performance thermal insulation. Such sustainable insulation systems represent an important aspect of future energy-efficient construction, where reducing operational energy consumption is closely linked to the environmental performance of building envelopes.

Several contributions investigated advanced nanomodification approaches. Park et al. [19] analysed the effect of graphene oxide on the geotechnical properties of soil and confirmed the significant potential of graphene-based nanomaterials for improving engineering performance and stability. The application of graphene and its derivatives in construction materials has recently become one of the fastest-growing research areas, owing to their exceptional mechanical, electrical, thermal, and durability-related properties [20,21]. A related contribution by Pivak et al. [22] examined lime-based mortars modified with graphene nanoplatelets for cultural heritage conservation. Their study demonstrated improvements in mechanical performance and durability while maintaining compatibility with historical masonry materials. Compatibility between restoration materials and original historic substrates remains a crucial requirement in cultural heritage conservation. At the same time, graphene-based additives offer promising opportunities to enhance the long-term durability and multifunctionality of traditional lime-based systems.

Camargo et al. [23] addressed sustainable binder technologies, investigating the drying sensitivity and mechanical reliability of alkali-activated binders with respect to particle size distribution and packing optimisation. Alkali-activated materials and geopolymers are increasingly recognised as promising low-carbon alternatives to conventional Portland cement systems due to their significantly reduced embodied CO_2_ emissions and excellent durability performance [24]. Nevertheless, challenges related to drying shrinkage, brittleness, precursor variability, and large-scale implementation still require further investigation [25].

The effect of glass-composite additives on cement-based products was also addressed in the Special Issue. The results confirm the growing importance of alternative mineral additives for clinker replacement and sustainable cement production. The increasing utilisation of supplementary cementitious materials, including glass powders, slags, calcined clays, and industrial by-products, is currently one of the key strategies for reducing the environmental footprint of cement-based materials [26,27,28].

Overall, the papers published in this Special Issue clearly demonstrate the rapidly growing importance of chemical additives and alternative admixtures in the research of sustainable construction materials. The presented studies confirm that advanced modifiers, recycled materials, nanotechnology, and alternative binder systems can significantly improve the performance and durability of construction composites while simultaneously reducing their environmental impact.

Future research should focus particularly on the long-term durability, lifecycle assessment, large-scale industrial applicability, and environmental impact of advanced sustainable materials under real service conditions. Additionally, greater attention should be devoted to multifunctional and smart construction materials, AI-assisted material design, self-sensing composites, energy-efficient production technologies, and the integration of nanomaterials into low-carbon cementitious systems. A successful transition towards sustainable and resilient construction technologies will therefore require interdisciplinary collaboration among materials scientists, chemists, civil engineers, and environmental researchers.

## Data Availability

All data related to the articles published in this Special Issue are available on request to the corresponding authors of the articles.

## References

[1] Scrivener K., John V.M., Gartner E. (2018). Eco-efficient cements: Potential economically viable solutions for a low-CO2 cement-based materials industry. Cem. Concr. Res..

[2] Marandi N., Shirzad S. (2025). Sustainable cement and concrete technologies: A review of materials and processes for carbon reduction. Innov. Infrastruct. Solut..

[3] Naseri H., Jahanbakhsh H., Hosseini P., Nejad F.M. (2020). Designing sustainable concrete mixture by developing a new machine learning technique. J. Clean. Prod..

[4] Mehta P.K., Monteiro P.J.M. (2014). Concrete: Microstructure, Properties, and Materials.

[5] Makhdoomi A.I., Prasad A., Kumar P., Purty S., Kumar P. (2025). Alkali-Activated Materials For Low-Carbon Construction: A Review. Int. J. Creat. Res. Thoughts.

[6] Asim N., Badiei M., Samsudin N.A., Mohammad M., Razali H., Soltani S., Amin N. (2022). Application of graphene-based materials in developing sustainable infrastructure: An overview. Compos. B Eng..

[7] Wei X.-X., Pei C., Zhu J.-H. (2024). Towards the large-scale application of graphene-modified cement-based composites: A comprehensive review. Constr. Build. Mater..

[8] Jirickova A., Lauermannova A.-M., Jankovsky O., Lojka M., Zaleska M., Pivak A., Pavlikova M., Pavlik Z. (2026). Graphene oxide as a nanoadditive in cementitious composites: A comparative study of portland and magnesium oxychloride cement binders. Constr. Build. Mater..

[9] Hiew S.Y., Teoh K.B., Wong H.S., Banthia N., Yoo D.-Y. (2026). Recent advances in low-carbon ultra-high-performance concrete: Materials, mechanisms, and sustainability perspectives. npj Mater. Sustain..

[10] Morrison J., Jauffret G., Galvey-Martez J.L., Glasser F.P. (2016). Magnesium-based cements for CO_2_ capture and utilisation. Cem. Concr. Res..

[11] Zhang Y., Song Z., Qi X., Zhang Y., Zhao S., Sun J., Guo M.-Z. (2025). Recycling red mud in magnesium oxychloride cement: A comparison study of organic acid modifiers enhancing water resistance and sustainability. J. Build. Eng..

[12] Jia Y., Zhang J., Zou Y., Guo Y., Li M., Zhang T., Cheeseman C. (2024). Development and applications of MgO-activated SiO_2_ system—Achieving a low carbon footprint: A review. Green Energy Resour..

[13] Lee S.-J., Shin H., Lee H.-N., Park S.-H., Kim H.-M., Park C.-G. (2024). Effect of Waste PET Fiber on the Mechanical Properties and Chloride Ion Penetration of Emergency Repair Concrete for Road Pavement. Materials.

[14] Mahmoudi A., Afshari H., Sarhadi H., Jabbarzadeh A. (2026). From waste to profit: An environmental, social, and governance (ESG) framework for integrating recycled plastic into sustainable concrete production. Transp. Res. Part E Logist. Transp. Rev..

[15] Abbas S.N., Qureshi M.I. (2025). Effect of recycled plastic aggregates on mechanical and durability properties of concrete: A review. Mater. Chem. Phys. Sustain. Energy.

[16] Skowron M., Lelek-Borkowska U., Kaczmarska K. (2026). Comparative Thermal and Fire Behavior of Rigid Polyurethane (PUR) and Polyisocyanurate (PIR) Foams Formulated with Recycled Poly(ethylene terephthalate) (PET) Polyols—Part 1. Materials.

[17] Pu M., Fang C., Zhou X., Wang D., Lin Y., Lei W., Li L. (2024). Recent Advances in Environment-Friendly Polyurethanes from Polyols Recovered from the Recycling and Renewable Resources: A Review. Polymers.

[18] Oumaima A.K., Naoual B., Latifa E., Abdellah Z. (2025). Sustainable aerogel blankets from recycled plastics and construction waste for high-performance thermal insulation. Hybrid Adv..

[19] Park K., Kim J.-H., Shin J., Lee H., Nam B.H. (2024). A Study on the Effect of Graphene Oxide on Geotechnical Properties of Soil. Materials.

[20] Shilar F.A., Ganachari S.V., Patil V.B. (2022). Advancement of nano-based construction materials-A review. Constr. Build. Mater..

[21] Vignes J., Ramesh B., Xavier J.R. (2025). A comprehensive review of materials, processing, and performance of nano-doped engineered geopolymer composites for construction applications. Case Stud. Constr. Mater..

[22] Pivak A., Pavlikova M., Zaleska M., Pavlik Z. (2024). Development and Characterization of Lime-Based Mortars Modified with Graphene Nanoplatelets. Materials.

[23] Camargo W.F., Segadães A.M., Cruz R.C.D. (2024). Assessing the Drying Sensitivity of Alkali-Activated Binders Through Mechanical Reliability: Effect of Particle Size and Packing. Materials.

[24] Bentegri I., De Campos M., Hasnaoui A., Reeb C., Albert-Mercier C., Davy C., Lambertin D. (2026). Durability of alkali-activated materials in cementitious leaching environments. Results Eng..

[25] Bumanis G., Vitala L., Bajare D., Dembovska L., Pundiene I. (2017). Impact of reactive SiO_2_/Al_2_O_3_ ratio in precursor on durability of porous alkali activated materials. Ceram. Int..

[26] Sharma J., Jang J.G., Bansal P.P. (2022). A comprehensive review on effects of mineral admixtures and fibers on engineering properties of ultra-high-performance concrete. J. Build. Eng..

[27] Ma S., Yang R., Lu Z., Lu H., Zhao W., Chen H., Liu M. (2025). Effects of different mineral admixtures on the properties of magnesium potassium phosphate cement mortar. Case Stud. Constr. Mater..

[28] Marchetti G., Rahhal V., Pavlik Z., Pavlikova M., Irassar E.F. (2020). Assessment of packing, flowability, hydration kinetics, and strength of blended cements with illitic calcined shale. Constr. Build. Mater..

